# Levamisole causes a transient increase in plasma creatinine levels but does not affect kidney function based on cystatin C

**DOI:** 10.1007/s00467-022-05547-9

**Published:** 2022-04-13

**Authors:** Floor Veltkamp, Arend Bökenkamp, Jeroen Slaats, Henrike Hamer, Antonia H. M. Bouts, on behalf of the LEARNS consortium

**Affiliations:** 1 Department of Pediatric Nephrology, Emma Children’s Hospital, Amsterdam University Medical Centers, University of Amsterdam, Meibergdreef 9, 1109 AZ Amsterdam, The Netherlands; 2 Department of Pediatric Nephrology, Emma Children’s Hospital, Amsterdam University Medical Centers, Free University, Amsterdam, The Netherlands; 3 Department of Clinical Chemistry, Amsterdam University Medical Centers, Free University, Amsterdam, The Netherlands

**Keywords:** Levamisole, Creatinine, Cystatin C, Estimated glomerular filtration rate

## Abstract

**Background:**

In pediatric patients treated with levamisole to prevent relapses of idiopathic nephrotic syndrome (INS), a transient and non-progressive rise in creatinine levels has been observed. It has been suggested that levamisole affects tubular secretion of creatinine. However, other potential mechanisms — nephrotoxicity and interference with the analytical assay for creatinine — have never been thoroughly investigated.

**Methods:**

In three steroid-sensitive nephrotic syndrome (SSNS) patients with elevated plasma creatinine levels, treated with levamisole 2.5 mg/kg every other day, serum cystatin C was determined. The glomerular filtration rate (GFR) was estimated using the full age spectrum for creatinine and the full age spectrum for cystatin C equations. Interference of levamisole with the enzymatic creatinine assay was tested using spare human plasma of different creatinine concentrations spiked with levamisole (4, 20, and 100 µM).

**Results:**

Three patients who received levamisole with elevated plasma creatinine levels had normal serum cystatin C levels and corresponding estimated GFR. There was no assay interference.

**Conclusion:**

Levamisole increases plasma creatinine levels, which is most probably due to impaired tubular secretion of creatinine since there was no assay interference and patients had normal eGFR based on serum cystatin C. However, interference of metabolites of levamisole could not be excluded. To monitor GFR, cystatin C in addition to creatinine should be used and be measured before and during levamisole use.

## Introduction

Levamisole has been increasingly used as a steroid-sparing agent in steroid-sensitive nephrotic syndrome (SSNS) in children. Although the exact mechanism of action in SSNS is still unclear, it is believed that it has immunomodulatory properties. Recent studies in children with frequently relapsing nephrotic syndrome (FRNS) or steroid-dependent nephrotic syndrome (SDNS) showed that levamisole is efficacious and safe to prevent relapses [1], even when administered on a daily basis [2]. In children, side effects are relatively uncommon and include leukopenia (3.7%), gastrointestinal upset (2.4%), and skin rash (1.5%), which are reversible after discontinuation of the treatment [3]. Additionally, ANCA positivity has been observed in up to 20% of children using levamisole [4].

Recently, Hoogenboom et al. described elevated plasma creatinine levels and a corresponding decrease in the estimated glomerular filtration rate (eGFR) calculated by the Schwartz equation [5] during levamisole treatment in a cohort of children with FRNS or SDNS [6]. This decrease in eGFR was not progressive and normalized after levamisole was discontinued. The authors speculated that levamisole might interfere with tubular handling of creatinine resulting in a rise in creatinine levels independent of GFR. Other potential mechanisms considered were interference of levamisole with the analytical assay for creatinine or transient nephrotoxicity.

In our hospital, levamisole has been extensively used for the prevention of relapses of SSNS. A similar trend of transient elevated plasma creatinine levels has been observed. In this report, we provide additional proof that the rise in plasma creatinine is indeed the result of levamisole-induced decreased tubular creatinine secretion, rather than assay interference or impaired kidney function.

## Methods

This study was conducted at the department of pediatric nephrology of Emma Children’s Hospital, Amsterdam University Medical Centers, Amsterdam, the Netherlands. All SSNS patients aged between 2 and 18 years who participated in the LEARNS study were screened for the use of levamisole for the prevention of relapses and documented creatinine and at least one cystatin C (cysC) measurements. The LEARNS study is an international, randomized, placebo-controlled trial investigating the efficacy and safety of adding levamisole to corticosteroids for the prevention of relapses of the first episode of INS [7]. The study was approved by the ethical committee of Amsterdam UMC, location AMC. Screened patients used levamisole in an open-label fashion after a relapse occurred. Relapses were treated according to the GPN protocol: 60 mg/m^2^/day until remission, followed by 40 mg/m^2^/alternate day for 4 weeks. After remission was achieved, levamisole (2.5 mg/kg every other day, maximum of 150 mg/dose) was initiated. A treatment duration of 12 months was intended. Reasons to discontinue levamisole included relapse, marked side effects, and prolonged remission (> 12 months). Estimated GFR was calculated from plasma creatinine or serum cysC using the age-based full age spectrum (FAS) equations [8].

Plasma creatinine concentrations were determined by a standardized enzymatic colorimetric method (Creatinine plus ver.2; Roche diagnostics, Mannheim, Germany) using the Cobas 8000 c702 module (Roche diagnostics, Mannheim, Germany). Serum cysC concentrations were determined by particle-enhanced immunonephelometry (N Latex Cystatin C; Siemens Healthineers, Erlangen, Germany) using the Atellica NEPH630 System (Siemens Healthineers, Erlangen, Germany).

To test the interference of levamisole with the creatinine analytical enzymatic assay, spare human blood samples from 9 adult patients with low (26–33 µmol/L) and high (105–108 µmol/L) plasma creatinine concentrations were spiked with increasing levamisole concentrations. Concentrations of 0, 4, 20, and 100 µM were obtained by diluting 200 mM levamisole stock solution with NaCl. All experiments were done at the Department of Clinical Chemistry of Amsterdam UMC, location VUmc.

## Results

### Patient presentation

Chart analysis identified six patients who received levamisole and in whom plasma creatinine and the corresponding eGFR were determined at every visit between 2018 and 2021. In all patients, plasma creatinine increased during treatment (data not shown). However, cysC had been measured at least once in three patients. CysC was determined in response to elevated plasma creatinine levels at the next visit and was only determined during levamisole treatment.

**Fig. 1:**
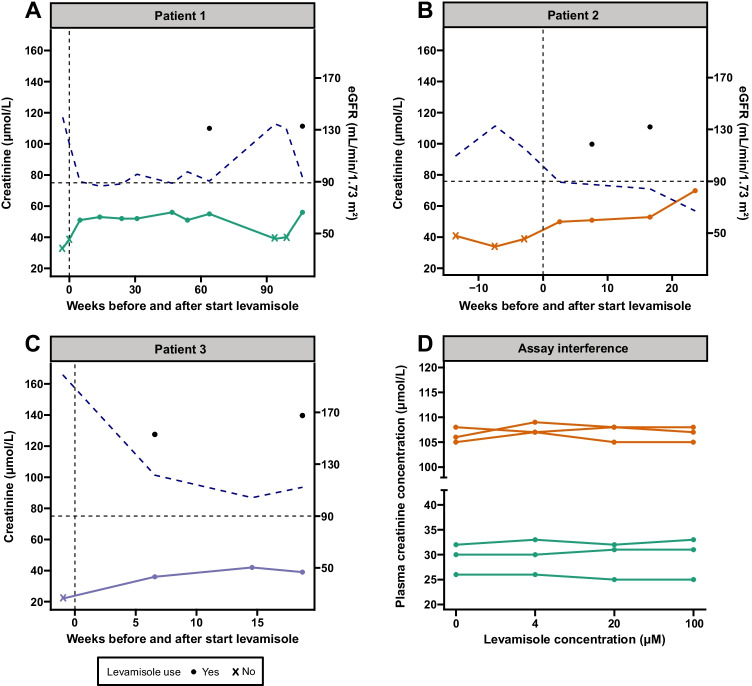
**a**–**c** Graphical display of the evolution of plasma creatinine and eGFR for three different patients. The colored solid lines represent the change in plasma creatinine concentrations (left y-axis) during the course of levamisole treatment, while the dashed blue lines represent the FAS_creat_ and the black dots show the FAS_cys_ (right y-axis). The vertical dashed line indicates the start of levamisole treatment, the horizontal dashed line indicates the cut-off value for impaired kidney function (90 mL/min/1.73 m^2^; right y-axis). In patient 1 (**a**), levamisole was restarted when she experienced a second relapse after discontinuation of levamisole. **d** Presentation of the measured plasma creatinine concentration by the enzymatic assay for low and high concentrations of levamisole

Patient 1 (female, age 6 years) received levamisole for 64 weeks during which she remained in remission. After 4 weeks, creatinine rose and remained stable between 50 and 55 µmol/L (FAS_creat_ around 90 mL/min/1.73 m^2^, while cysC was at 0.80 mg/L (FAS_cys_ 101 mL/min/1.73 m^2^). When levamisole was discontinued, plasma creatinine decreased to 39 µmol/L. However, the patient experienced a new series of relapses and levamisole was started again. Four weeks after restart, plasma creatinine rose from 40 to 56 µmol/L, again with normal cysC levels (0.79 mg/L) and corresponding FAS_cys_ (116 mL/min/1.73 m^2^) (Fig. 1a).

In patient 2 (male, age 5 years), creatinine had risen from 38 to 49 µmol/L within 3 weeks after introduction of levamisole and continued to rise to a maximum of 69 µmol/L. When FAS_creat_ had dropped to 78 mL/min/1.73 m^2^, cysC was determined at 0.89 mg/L, corresponding to a FAS_cys_ of 102 mL/min/1.73 m^2^ (Fig. 1b). Therefore, levamisole was continued.

Patient 3 (male, age 5 years) was in partial remission when levamisole was started. Prednisolone was gradually tapered over the course of levamisole treatment. Before the start of levamisole, creatinine had been 22 µmol/L, but increased to 36 µmol/L and 42 µmol/L after 4 and 14 weeks, respectively. During levamisole treatment, FAS_creat_ (101 mL/min/m^2^) and FAS_cys_ (125 mL/min/m^2^) remained above the threshold for impaired kidney function (Fig. 1c).

### Assay interference

There was no interference of levamisole with the enzymatic creatinine assay. With increasing levamisole concentrations, serum creatinine concentrations did not deviate from baseline measurements, with low and high plasma creatinine concentrations showing minimal deviations up to 4% and 2.8%, respectively (Fig. 1d).

## Discussion

In line with Hoogenboom et al. [6], we observed an increase in plasma creatinine levels in three children during levamisole treatment. Yet, this was not associated with an impaired eGFR when calculated from cysC levels. When levamisole was discontinued, creatinine levels returned to normal. Additionally, levamisole did not interfere with the analytical enzymatic assay for creatinine in plasma.

Three potential mechanisms for the elevated serum creatinine were considered: (1) nephrotoxicity, (2) interference with the analytical assay, and (3) impaired tubular secretion. Hoogenboom et al. considered nephrotoxicity of levamisole unlikely as the rise in plasma creatinine was not progressive and independent of dose and duration of treatment. This is supported by our finding that, based on cysC levels, eGFR was not impaired in our three patients. In line with these findings, inulin clearance did not change in a study in dogs treated with levamisole [9].

To our knowledge, this is the first study that shows that levamisole does not interfere with the enzymatic assay to measure creatinine in plasma, not even at supra-physiological concentrations. In a study in children receiving alternate day levamisole, the C_max_ was 438 ng/mL (2.36 µM) [10]. Particularly, there was no change at low and high creatinine concentrations. As children with INS are young (peak incidence 2–6 years) and have a normal kidney function, reference intervals for creatinine concentrations lie between 18 and 54 µmol/L [11]. However, levamisole has a short half-life of 2.6 h [10] and potential assay interference with one of its metabolites, i.e., aminorex and p-hydroxylevamisole, cannot be excluded. Having eliminated nephrotoxicity and — presumably — assay interference as potential mechanisms of creatinine elevation by levamisole, interference with the tubular secretion of creatinine remains as the most probable explanation.

Creatinine has a low molecular weight (113 Da), is not protein-bound, and is freely filtered across the glomerular basement membrane. Although chiefly considered a marker for GFR, creatinine is also excreted by the renal tubules [12]. Tubular secretion is variable and its rate is inversely related to GFR. It can be inhibited by a number of drugs through inhibition of organic cation transporters (OCTs), of which cimetidine, trimethoprim, and fenofibrate are well-known. There is limited evidence whether levamisole is an OCT inhibitor: one study found an inhibitory effect on the rOCT1 in rat hepatocytes [13]. However, whether this OCT is present in humans is uncertain.

CysC is a low-molecular weight protein (13 kDa), which has evolved as an alternative to plasma creatinine for the monitoring of kidney function [12]. Serum cysC concentrations are inversely related to GFR. CysC is produced at a constant rate by nearly all nucleated cells. Like other low-molecular weight proteins, it is freely filtered across the glomerular membrane and almost entirely reabsorbed and metabolized in the proximal tubule. Therefore, only trace amounts can be found in the urine. There are no indications that cysC is secreted in the kidney tubule; still, there is some breakdown in the liver, which is relevant in severe kidney failure only and has not been linked to any drug therapy [14]. A relevant interaction in the setting of INS is high-dose glucocorticoid treatment, since this increases cysC synthesis and results in an underestimation of GFR [15].

In conclusion, our findings support the concept that levamisole increases plasma creatinine by interfering with the tubular secretion of creatinine and argue against nephrotoxicity and assay interference. Although an increased plasma creatinine level in children using levamisole should not prompt clinical concern, we recommend using additional cysC-based equations to confirm normal kidney function. In case of decreased creatinine-based eGFR and in the absence of an abnormal cysC-based eGFR, levamisole should not be discontinued.

## Data Availability

The datasets generated during and/or analysed during the current study are available from the corresponding author on reasonable request.

## Code availability

The R and GraphPad syntaxes used for the development of the graphs are available upon request.
