# Correction to: Levamisole causes a transient increase in plasma creatinine levels but does not affect kidney function based on cystatin C

**DOI:** 10.1007/s00467-022-05622-1

**Published:** 2022-06-28

**Authors:** Floor Veltkamp, Arend Bökenkamp, Jeroen Slaats, Henrike Hamer, Antonia H. M. Bouts, on behalf of the LEARNS consortium

**Affiliations:** 1 Department of Pediatric Nephrology, Emma Children’s Hospital, Amsterdam University Medical Centers, University of Amsterdam, Meibergdreef 9, 1109 AZ Amsterdam, The Netherlands; 2 Department of Pediatric Nephrology, Emma Children’s Hospital, Amsterdam University Medical Centers, Free University, Amsterdam, The Netherlands; 3 Department of Clinical Chemistry, Amsterdam University Medical Centers, Free University, Amsterdam, The Netherlands

**Correction to** **:** **Pediatr Nephrol**

The original version of this article unfortunately contained a mistake. During the process of typesetting, the institutional author attribution “on behalf of the LEARNS consortium” was left out of the author group. The correct attribution is as shown below. The publisher apologizes for this mistake. The original article was updated.

